# The effects of mouth rinsing and gargling with mouthwash containing povidone-iodine and hydrogen peroxide on the cycle threshold value of Severe Acute Respiratory Syndrome Coronavirus 2: A randomized controlled trial of asymptomatic and mildly symptomatic patients

**DOI:** 10.12688/f1000research.110843.1

**Published:** 2022-11-01

**Authors:** Lilies Dwi Sulistyani, Vera Julia, Andrianto Soeprapto, Rumartha Putri Swari, Febriadi Rosmanato, Budi Haryanto, Cahyarini Cahyarini, Rinaldi Panjaitan, Diah Ayu Maharani

**Affiliations:** 1 Oral and Maxillofacial Surgery, Faculty of Dentistry, Universitas Indonesia, Central Jakarta, DKI Jakarta, 10430, Indonesia; 2 Clinical Microbiology, Persahabatan Central General Hospital, East Jakarta, DKI Jakarta, 13230, Indonesia; 3 Department of Preventive and Public Health Dentistry, Faculty of Dentistry, Universitas Indonesia, Central Jakarta, DKI Jakarta, 10430, Indonesia

**Keywords:** mouthwash, severe acute respiratory syndrome coronavirus, povidone iodine

## Abstract

**Background:** Coronavirus disease 2019 can spread rapidly. Surgery in the oral cavity poses a high risk of transmission of severe acute respiratory syndrome coronavirus 2. The American Dental Association and the Centers for Disease Control and Prevention recommend the use of mouthwash containing 1.5% hydrogen peroxide (H _2_O _2_) or 0.2% povidone iodine (PI) to reduce the viral load in the upper respiratory tract and decrease the risk of transmission. The aim of the present study was to analyze the effect of mouth rinsing and gargling with mouthwash containing 1% PI, 0.5% PI, 3% H _2_O _2_, or 1.5% H _2_O _2_ and water on the cycle threshold (CT) value obtained by real-time reverse transcription polymerase chain reaction (RT-PCR).

**Methods:** This study is a randomized single blind controlled clinical trial which has been registered in the International Standard Randomized Controlled Trial Number (ISRCTN) registry on the 3 ^rd^ February 2022 (Registration number: ISRCTN18356379). In total, 69 subjects recruited from Persahabatan General Hospital who met the inclusion criteria were randomly assigned to one of four treatment groups or the control group. The subjects were instructed to gargle with 15 mL of mouthwash for 30 s in the oral cavity followed by 30 s in the back of the throat, three times per day for 5 days. CT values were collected on postprocedural days 1, 3, and 5.

**Results:** The results of the Friedman test significantly differed among the groups (n=15). The CT values increased from baseline (day 0) to postprocedural days 1, 3, and 5.

**Conclusions:** Mouth rinsing and gargling with mouthwash containing 1% PI, 0.5% PI, 3% H _2_O _2_, or 1.5% H _2_O _2_ and water increased the CT value.

## 1. Introduction

Coronavirus disease 2019 (COVID-19) spreads quickly and deadly and, thus, has been especially challenging to healthcare workers. ^1^ COVID-19, which is caused by severe acute respiratory syndrome coronavirus 2 (SARS-CoV-2) infection of the respiratory tract, was first reported in Wuhan, China in December 2019 and declared a pandemic on March 11, 2020. ^2^^–^^4^ According to the World Health Organization, SARS-CoV-2 accounted for 262 million infections and 5.2 million deaths globally as of December 1, 2021. ^5^ The average incubation period of SARS-CoV-2 is about 3–9 days. ^6^ Clinical manifestations appear after the incubation period and vary from asymptomatic to severe respiratory disease and life-threatening multiple organ failure. ^1^^,^^7^

Up to 80% of cases exhibit mild localized symptoms in the upper respiratory tract (URT) accompanied by non-specific symptoms, especially fever and cough. ^6^^,^^8^^,^^9^ Virus transmission in about 44% of cases occurs before the onset of symptoms. ^10^ About 18% of cases are asymptomatic but can transmit the virus to others. ^6^ It is difficult to distinguish between truly asymptomatic and pre-symptomatic patients because of the lack of visible symptoms. ^11^ Asymptomatic patients and those with mild symptoms greatly contribute to the transmission of SARS-CoV-2 because of the lack of awareness of an active infection, reluctance to seek medical care, and poor understanding of transmission prevention. ^12^

SARS-CoV-2 is mainly transmitted by inhalation of respiratory droplets or contact with contaminated surfaces. ^7^^,^^13^ SARS-CoV-2 rapidly replicates in the URT, producing large numbers of pathogenic progeny at an early stage of disease development that can be transmitted by respiratory droplets. ^1^^,^^14^ Further replication in the lower respiratory tract leads to the development of lung disease. ^15^^,^^16^ Saliva contains high amounts of SARS-CoV-2 that enters through the lower respiratory tract, URT, or infected salivary glands and acts as a potential source of virus transmission in the oral cavity. ^17^^–^^20^

Reducing the amount of SARS-CoV-2 in the URT and oral cavity in the early stages of disease is important to prevent virus transmission and reduce the severity and progression of disease. ^18^^,^^21^^,^^22^ Various active ingredients of mouthwash have virucidal activities that disrupt the lipid envelope of the virus. ^23^ Active ingredients of mouthwash recommended before medical procedures in the oral cavity include 21%–26% ethanol with essential oils, chlorhexidine, povidone iodine (PI), hydrogen peroxide (H _2_O _2_), cetylpyridinium chloride, chlorinated water, and hypertonic saline. ^23^^–^^25^ The American Dental Association (ADA) and the Centers for Disease Control and Prevention (CDC) recommend gargling with mouthwash containing 0.2% PI or 1.5% H _2_O _2_ before medical procedures in the oral cavity because SARS-CoV-2 is susceptible to oxidation. ^26^^,^^27^ Current recommendations include mouth rinsing and gargling with mouthwash for 30 s in the oral cavity followed by 30 s in the back of the throat. ^24^

The objective of this study was to semi-quantitatively evaluate the effect of mouth rinsing and gargling with mouthwash containing various concentrations of PI and H _2_O _2_ on the amount of SARS-CoV-2 in the URT using the cycle threshold (CT) value during real-time reverse transcription polymerase chain reaction (RT-PCR) in asymptomatic and mildly symptomatic patients.

## 2. Methods

### 2.1 Study design

The cohort of this single-blind randomized controlled trial included four intervention groups and one control group. The study protocol was approved by the Health Research Ethics Committee of Persahabatan Central General Hospital (CGH) with registration number: 68/KEPK-RSUPP/06/2021. This study has been retrospectively registered in the International Standard Randomized Controlled Trial Number (ISRCTN) registry on the 3 ^rd^ February 2022 with registered number ISRCTN18356379 ( https://doi.org/10.1186/ISRCTN18356379).

### 2.2 Sample selection

In total, 69 patients infected with SARS-CoV-2 were recruited from Persahabatan CGH, a national COVID-19 referral center hospital, from July to September 2021. SARS-CoV-2 infection was confirmed by RT-PCR. The sample size was calculated using G*Power (RRID:SCR_013726) 3.1.9.2 software ( https://www.psychologie.hhu.de/arbeitsgruppen/allgemeine-psychologie-und-arbeitspsychologie/gpower). The inclusion criteria were age 19–60 years, CT values ≤ 30, asymptomatic or mild symptoms, and diagnosis of COVID-19 within 3 days prior to recruitment. The exclusion criteria consisted of refusal to participate, comorbid disease, thyroid disease, pregnancy, routine use lithium drugs, radioactive iodine treatment, and allergy to PI and H _2_O _2_. All subjects signed an informed consent form after being provided with information regarding the study objective and possible risks and benefits of gargling with mouthwash. The subjects were randomly assigned by one researcher to one of the four treatment groups or the control group using a simple randomization method where each research subject was assigned to a group with specific order: 1% PI, 3% H _2_O _2_, control, 0.5% PI, and 1.5% H _2_O _2_ consecutively. This study is single blinded where only the research subjects were blinded from their group allocation.

### 2.3 Intervention

The subjects were instructed on how to rinse and gargle with mouthwash via video conference and supplied with repackaged mouthwash. The 1% PI group rinsed their mouth with BETADINE ^®^ Mouthwash and Gargle solution (Napp Pharmaceuticals Ltd., United Kingdom). The 3% H _2_O _2_ group rinsed their mouth with *OneMed™* solution (Inti Medicom Retailindo, Indonesia). The group treated with 0.5% iodine peroxide and 1.5% H _2_O _2_ rinsed their mouth with a diluted solution of 1% PI BETADINE ^®^ Mouthwash and Gargle solution and 3% H _2_O _2_ OneMed *™* added with sterile distilled water in accordance with the formula Volume _1_ × Concentration _1_ = Volume _2_ × Concentration _2_. The control group was instructed to rinse their mouth with AQUA *™* mineral water (Danone, France). The subjects were instructed to rinse their mouth with 15 mL of mouthwash for 30 s in the oral cavity followed by gargling 30 s in the back of throat three times per day for 5 days. Mouth rinsing and gargling with mouthwash were conducted in a self-isolation room and monitored via video conference.

### 2.4 Measurement

Samples were collected with oropharyngeal and nasopharyngeal swabs using a disposable virus sampling tube (Baicare Biotechnology Co., Ltd., China) by a trained staff member of Persahabatan CGH on postprocedural days 1, 3, and 5 after gargling with mouthwash. The samples were appropriately packaged, labeled, and sent to the Department of Microbiology for RT-PCR analysis. The specimens were vortexed with an LMS ^®^ UZUSIO VTX-3000L vortex mixer (LMS Co., Ltd., Japan) for 20 s and allowed to stand for 15 min. Then, 250 μL of MagNA Pure 96 extraction reagent (Roche Life Science, Germany) were loaded into the cartridge and mixed with 200 μL of the specimen. The cartridge was loaded into the Rosche MagNA Pure 96 instrument for sample extraction. The reaction mix of the mBioCoV-19 RT-PCR Kit (Bio Farma, Indonesia) was used to detect the open reading frame 1b and RNA-dependent RNA polymerase genes. In brief, 15 μL of reaction mix were added to each well and mixed with 5 μL of the extracted specimen. CT values were obtained automatically with an Exicycler™ 96 (Ver.4) (RRID:SCR_022144) Real-Time Quantitative Thermal Block (Bioneer Corporation, South Korea) ( https://us.bioneer.com/products/instrument/Exicycler96_V4-overview.aspx) upon detection of SARS-CoV-2 genetic material.

### 2.5 Statistical analysis

CT values of the open reading frame 1b target gene were analyzed using IBM SPSS Statistics for Windows, version 22.0. (IBM Corporation, USA) (RRID:SCR_016479) ( https://www.ibm.com/products/spss-statistics). The data were not normally distributed; thus a nonparametric test was used for analysis. Repeated measurements of each group were analyzed using the Friedman nonparametric test. Comparisons between groups from baseline (day 0) to postprocedural days 1, 3, and 5 were conducted using the Kruskal–Wallis nonparametric test. A probability ( *p*) value of <0.05 was considered statistically significant.

## 3. Results

**Figure 1.**
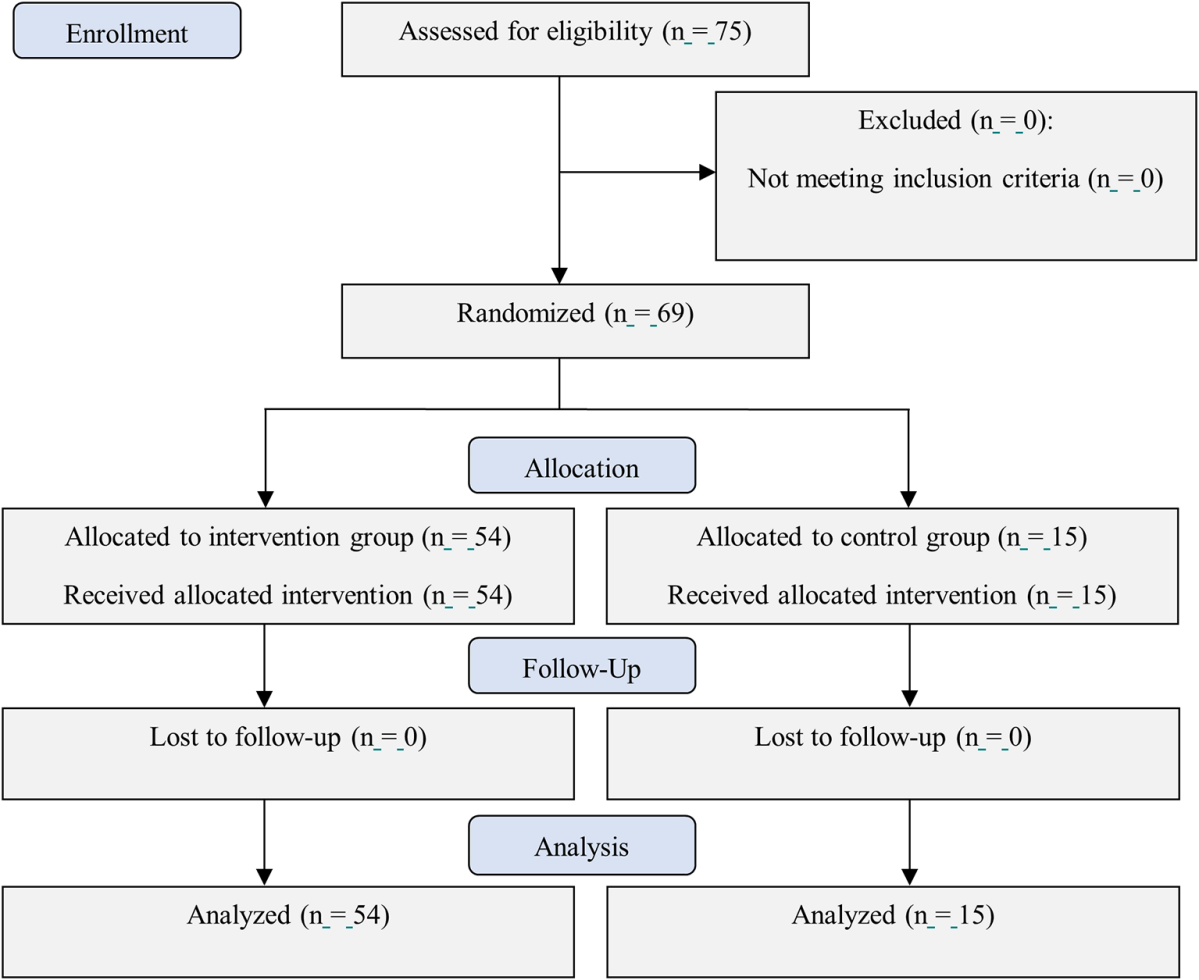
CONSORT flow diagram of this study.

The total size estimation was 75 patients with n=15 for each group. However, due to the significant decrease in new COVID-19 cases in Indonesia, only 69 patients were recruited from July to September 2021, as it was difficult to recruit subjects who met the inclusion criteria ( Figure 1). Numbers of participants for each group were 1% PI = 15, 0.5% PI = 12, 3% H _2_O _2_ = 15, 1.5% H _2_O _2_ = 12, and control = 15.

Of the 69 patients, 39 (56.5%) were male and 30 (43.5%) were female. The average age of the subjects was 32.8 (range, 25–44) years. Cough (66.7%) was the most common early symptom of COVID-19 ( Table 1). As shown in Table 2, the mean CT values increased in each group from baseline (day 0) to postprocedural days 1, 3, and 5. The results of the Friedman test showed significant differences in CT values among the groups. *Post-hoc* analysis ( Table 3) showed significant differences in most of the CT values with the exceptions of between days 1 and 3 in the 0.5% PI and 3% and 1.5% H _2_O _2_ groups and between days 3 and 5 in the 3% H _2_O _2_ and control groups. Comparisons of CT values among the groups using the Kruskal–Wallis test showed no significant differences due to the increases in CT values of each group.

**Table 1.** Demographic data.

Sex	n	Percentage (%)
Male	39	56.5
Female	30	43.5
**Age (years)**		
19–25	14	20.3
25–44	45	65.2
45–60	10	14.5
**Early symptoms**		
Fever	38	55.1
Cough	46	66.7
Fatigue	41	59.4
Sore throat	29	42.0
Runny nose	40	58.0
Headache	38	55.1
Digestive disorders (diarrhea, nausea, vomiting)	25	36.2
Anosmia	30	43.5
Ageusia	18	26.1

**Table 2.** Comparisons of cycle threshold (CT) values among the povidone iodine, hydrogen peroxide, and control groups ( *p<0.05).

	CT Value								*p* (Friedman test)
	Baseline (Day 0)		Day 1		Day 3		Day 5		
	Mean	SD	Mean	SD	Mean	SD	Mean	SD	
**PI, 1%**	23.974	4.017	29.105	6.041	34.035	5.765	36.879	4.406	**0.001** *
**PI, 0.5%**	24.153	4.856	32.907	8.621	35.319	7.476	37.781	3.984	**0.001** *
**H**_**2**_ **O**_**2**_ **3%**	23.132	3.806	33.097	5.783	35.679	5.590	37.861	3.310	**0.001** *
**H**_**2**_ **O**_**2**_ **1.5%**	25.405	3.639	32.390	6.627	35.639	5.757	38.531	1.960	**0.001** *
**Control**	24.685	3.737	31.147	6.351	35.313	5.689	36.773	6.369	**0.001** *
***p* (Kruskal–Wallis test)**	**0.562**		**0.307**		**0.850**		**0.969**		

* *p* < 0.05.

**Table 3.** Wilcoxon *post-hoc* analysis of the Friedman test.

	Baseline–Day 1	Baseline–Day 3	Baseline–Day 5	Days 1–3	Days 1–5	Days 3–5
PI, 1%	0.006 *	0.001 *	0.001 *	0.026 *	0.004 *	0.037 *
PI, 0.5%	0.005 *	0.004 *	0.002 *	0.093	0.012 *	0.043 *
H _2_O _2_ 3%	0.001 *	0.001 *	0.001 *	0.158	0.019 *	0.114
H _2_O _2_ 1.5%	0.012 *	0.003 *	0.002 *	0.066	0.008 *	0.028 *
Control	0.005 *	0.001 *	0.001 *	0.013 *	0.003 *	0.176

* *p* < 0.05.

## 4. Discussion

SARS-CoV-2 infection can spread rapidly and cause severe morbidity and mortality. ^28^ The oral cavity and URT have high viral loads and are potential sources of SARS-CoV-2. ^29^ Kim *et al*. ^30^ found that viral shedding was high in the URT from the prodromal phase to day 5 after symptom onset. Yoon *et al*. ^19^ found that the viral load was greater in the saliva than the oropharynx in the early stages of disease. A high viral load in saliva can originate from the respiratory tract or secretions from infected salivary glands. ^18^ Chen *et al*. ^20^ reported the expression of angiotensin converting enzyme 2, a cell receptor for SARS-CoV-2, in the salivary glands, suggesting possible SARS-CoV-2 infection of the salivary glands.

Reducing the amounts of SARS-CoV-2 in the URT and oral cavity is important to prevent virus transmission. ^21^^,^^22^ The ADA and the CDC recommend preprocedural mouth rinsing and gargling with mouthwash containing H _2_O _2_ or PI because SARS-CoV-2 is susceptible to oxidation. ^26^^,^^27^ PI consists of iodine and the water-soluble polymer polyvinylpyrrolidone. Iodine released from polyvinylpyrrolidone penetrates microorganisms and causes oxidation of amino acids and nucleic acids, resulting in disruption of metabolic pathways and cell membranes. ^17^^,^^25^ Frank *et al*. ^21^ claimed that mouthwash containing 2.5% PI is safe to use in the oral cavity for up to 5 months.

An *in vitro* study by Eggers *et al*. ^31^ reported that 0.023% PI exhibited virucidal activities against betacoronaviruses, including SARS-CoV and Middle East respiratory syndrome–related coronavirus (MERS-CoV), after contact for 15 s. The use of mouthwash containing 1% PI to reduce the load of SARS-CoV-2 was also confirmed by an *in vitro* study conducted by Anderson *et al*. ^3^ which reported that 1% PI reduced the load of SARS-CoV-2 by more than 99.99% or more than 4log _10_ after 30 s of contact. An *in vitro* study by Hassandarvish *et al*. ^32^ reported that 1% PI reduced of the load of SARS-CoV-2 by more than 5log _10_ after exposure for 15, 30, and 60 s. An *in vitro* study by Bidra *et al*. ^29^ found that PI at 0.5%, 1.25%, and 1.5% fully inactivated SARS-CoV-2 after contact for 15 and 30 s. The results of the present study showed that mouth rinsing and gargling with mouthwash containing 0.5% or 1% PI increased the CT values on postprocedural days 1, 3, and 5.

The virucidal action of H _2_O _2_ involves the release of oxygen free radicals that disrupt lipid membranes. ^17^ O’Donnell *et al*. ^23^ suggested that the target of H _2_O _2_ is the lipid envelope of SARS-CoV-2. The swine flu, rubella, rabies, corona, and influenza viruses are also sensitive to H _2_O _2_. ^24^^,^^33^ Caruso *et al*. ^33^ claimed that mouthwash containing 3% H _2_O _2_ was safe for mucous membranes after 6 months of use. An *in vitro* study by Kampf *et al*. ^34^ reported that H _2_O _2_ at a concentration of at least 0.5% effectively inactivated SARS-CoV and MERS-CoV on the surface of inanimate objects in 1 min. The results of the present study showed that mouth rinsing and gargling with mouthwash containing 1.5% and 3% H _2_O _2_ increased the CT values on postprocedural days 1, 3, and 5. In contrast with an *in vitro* study by Bidra *et al*., ^29^ which reported that mouthwash containing 1.5% and 3% H _2_O _2_ had minimal virucidal activity after contact for 30 s, Gottsauner *et al*. ^35^ showed that mouth rinsing and gargling with mouthwash containing 1% H _2_O _2_ in the mouth and back of the throat for 30 s did not reduce the load of SARS-CoV-2. The difference in the results of the present study and the report by Gottsauner *et al*. ^35^ was likely due to differences in H _2_O _2_ concentrations.

Vergara-Buenaventura *et al*. ^24^ recommended mouth rinsing and gargling with mouthwash for 30 s in the oral cavity and 30 s at the back of the throat. The control group in the present study gargled with water, which surprisingly increased the CT values on postprocedural days 1, 3, and 5. Flushing the URT with fluids can clear excess mucus and mechanically reduce the viral load in the respiratory tract with impaired mucociliary function. ^36^^–^^38^ A study by Koparal *et al*. ^39^ found a delay in mucociliary clearance time in COVID-19 patients as compared to healthy individuals. Robinot *et al*. ^40^ reported that SARS-CoV-2 infection in ciliated epithelial cells causes loss of ciliary motility, short cilia deformity, and impaired mucociliary clearance, thereby increasing the spread of SARS-CoV-2 in the respiratory tract and increasing the risk of secondary infection in COVID-19 patients. Whirling water can mechanically wash out the virus and virus-infected cells from the oral cavity and pharynx. ^41^ A study by Satomura *et al*. ^41^ reported that mouth rinsing and gargling with tap water three times per day effectively reduced the incidence of URT infections by 36%.

Several current guidelines regarding the management of discharge of COVID-19 patients are based on the timing from onset of symptoms and a CT value > 30. ^42^ CT values are often associated with the risk of SARS-CoV-2 transmission. ^43^ Patients with high CT values are reportedly incapable of transmitting infectious virus particles. ^44^ A study by Hiroi *et al*. ^45^ reported that a CT value >30 indicates a very low infectious virus titer and a lower risk of infecting others. Scola *et al*. ^46^ concluded that patients with CT values ≥ 34 are incapable of transmitting SARS-CoV-2 and could be discharged from the infectious disease ward. The entire cohort of the present showed an increase in mean CT score of 34 on day 3 after gargling.

This study may be limited by its limited and uneven sample number due to the significant decrease in new COVID-19 cases in Indonesia, thus only 69 patients were found to fulfil the inclusion criteria during the recruitment period, with two groups (0.5% PI and 1.5% H _2_O _2_) had only 12 samples per group where others had 15 per group.

## 5. Conclusion

Mouth rinsing and gargling with mouthwash containing 1% PI, 0.5% PI, 3% H _2_O _2_, or 1.5% H _2_O _2_ and water increased the CT values. The results of this study suggest that rinsing for 30 s in the oral cavity and 30 s at the back of the throat three times per day for at least 3 days preoperatively as a new preoperative protocol in oral and maxillofacial surgery and other medical procedures in the oral cavity.

## Data availability

### Underlying data

Zenodo: [Effects of Mouthrinsing and Gargling to CT Values of SARS CoV-2 DATASET]. https://doi.org/10.5281/zenodo.6358988 [Version 1.0].

The project contains the following underlying data:

‐ [informed-consent-back.jpg] (informed consent form page 2).
‐ [informed-consent-front.jpg] (informed consent form page 1).
‐ [Metadata (Eng).xlsx] (metadata to read the data file)
‐ [Reseach_Data_Revised.pdf] (raw data of each research subject)
‐ [research-information-form.jpg] (information sheet for research participants)
‐ [research-subject-screening-form.jpg] (form filled by the research subject on screening)
‐ [subject-consent-form.jpg] (consent form signed by research participants)

## Reporting guidelines

Zenodo: CONSORT checklist for ‘The effects of mouth rinsing and gargling with mouthwash containing povidone-iodine and hydrogen peroxide on the cycle threshold value of Severe Acute Respiratory Syndrome Coronavirus 2: A randomized controlled trial of asymptomatic and mildly symptomatic patients’. https://doi.org/10.5281/zenodo.6409184 [Version 1].

Data are available under the terms of the Creative Commons Attribution 4.0 International (CC BY 4.0).

## Author contributions

• Lilies Dwi Sulistyani conceived and designed the analysis, verified the analytical methods, gaining resources, supervised the study, in charge of overall direction and planning, wrote and revised the paper
• Vera Julia conceived and designed the analysis, verified the analytical methods, supervised the study, wrote and revised the paper
• Andrianto Soeprapto performed the experiment and analysis, drafted and revised the paper
• Rumartha Putri Swari performed the experiment and analysis, drafted the paper
• Febriadi Rosmanato performed the experiment and analysis, drafted the paper
• Budi Haryanto verified the analytical methods, gaining resources, supervised the study
• Cahyarini verified the analytical methods, supervised the study
• Rinaldi Panjaitan verified the analytical methods, supervised the study
• Diah Ayu Maharani designed and verified the statistics
