# Supplementary material for: The effects of mouth rinsing and gargling with mouthwash containing povidone-iodine and hydrogen peroxide on the cycle threshold value of Severe Acute Respiratory Syndrome Coronavirus 2: A randomized controlled trial of asymptomatic and mildly symptomatic patients
Source: F1000Res. 2024 Jul 1;11:1238. Originally published 2022 Nov 1. [Version 2] doi: 10.12688/f1000research.110843.2 (PMC11234079; doi:10.12688/f1000research.110843.2)
Supplement: Peer review history

# Reviewer report: Reviewer response for version 2

**DOI:** 10.5256/f1000research.165444.r297806

**Published:** 2024-07-09

**Authors:** Muhammad Ruslin

**Affiliations:** 1 Department of Oral and Maxillofacial Surgery, Faculty of Dentistry, Hasanuddin University, Makassar, Indonesia

Thank you for your revision on the article. Currently the conclusions are aligned with the data and the presentation looks clearer. I now approve the current version to be indexed.

Is the work clearly and accurately presented and does it cite the current literature?

Yes

If applicable, is the statistical analysis and its interpretation appropriate?

I cannot comment. A qualified statistician is required.

Are all the source data underlying the results available to ensure full reproducibility?

Yes

Is the study design appropriate and is the work technically sound?

Yes

Are the conclusions drawn adequately supported by the results?

Partly

Are sufficient details of methods and analysis provided to allow replication by others?

Yes

Reviewer Expertise:

dentistry, oral and maxillofacial surgery

I confirm that I have read this submission and believe that I have an appropriate level of expertise to confirm that it is of an acceptable scientific standard.

# Reviewer report: Reviewer response for version 2

**DOI:** 10.5256/f1000research.165444.r297805

**Published:** 2024-07-08

**Authors:** Rosalina Tjandrawinata

**Affiliations:** 1 Universitas Trisakti, West Jakarta, Jakarta, Indonesia

I read the revised version of the article and approved the revision. This article will be useful for standard operating procedures in oral and dental treatment.

Is the work clearly and accurately presented and does it cite the current literature?

Yes

If applicable, is the statistical analysis and its interpretation appropriate?

Partly

Are all the source data underlying the results available to ensure full reproducibility?

Yes

Is the study design appropriate and is the work technically sound?

Yes

Are the conclusions drawn adequately supported by the results?

Partly

Are sufficient details of methods and analysis provided to allow replication by others?

Yes

Reviewer Expertise:

Dental materials

I confirm that I have read this submission and believe that I have an appropriate level of expertise to confirm that it is of an acceptable scientific standard.

# Reviewer report: Reviewer response for version 1

**DOI:** 10.5256/f1000research.122496.r257581

**Published:** 2024-04-02

**Authors:** Rosalina Tjandrawinata

**Affiliations:** 1 Universitas Trisakti, West Jakarta, Jakarta, Indonesia

I learn the authors’ paper "The effects of mouth rinsing and gargling with mouthwash containing povidone-iodine and hydrogen peroxide on the cycle threshold value of Severe Acute Respiratory Syndrome Coronavirus 2: A randomized controlled trial of asymptomatic and mildly symptomatic patients" is very interesting and add more information for COVID-19-related cases.

 However, there are some questions about the effectivity of different concentration, which would be very useful. Statistical analysis should be taken to analyse which mouthwash is the most effective mouthwash for the cases.

 The conclusion is “that rinsing for 30 s in the oral cavity and 30 s at the back of the throat three times per day for at least 3 days preoperatively as a new preoperative protocol in oral and maxillofacial surgery and other medical procedures in the oral cavity”; while the statistics (Table 3) showed that both concentration of Hydrogen Peroxide and PI 0.5% mouthwash do not have significant difference between 1 day and 3 days and that CT value is already > 30.

 So, that 1-day rinsing would be effective enough for reducing the virus. It is quite interesting that your data (Table 2) showed PI 0.5% increase CT value similar or more than PI 1%.

 The most interesting condition is rinsing with mineral water (control group) is also rising the CT value, similar to the treated group. So, should we use mouth rinsing instead of mineral water if we can get similar result? It should be reconfirmed by authors. It will be very useful if the authors compare the difference among the mouth rinses and the control group.

Is the work clearly and accurately presented and does it cite the current literature?

Yes

If applicable, is the statistical analysis and its interpretation appropriate?

Partly

Are all the source data underlying the results available to ensure full reproducibility?

Yes

Is the study design appropriate and is the work technically sound?

Yes

Are the conclusions drawn adequately supported by the results?

Partly

Are sufficient details of methods and analysis provided to allow replication by others?

Yes

Reviewer Expertise:

Dental materials

I confirm that I have read this submission and believe that I have an appropriate level of expertise to confirm that it is of an acceptable scientific standard, however I have significant reservations, as outlined above.

# Response

**Published:** 2024-04-22

**Authors:** Lilies Dwi Sulistyani

**Affiliations:** University of Indonesia, Indonesia

Thank you to Rosalina Tjandrawinata. We appreciate your review and questions.

 Mouth rinsing and gargling for 1 day shows effectiveness in reducing the number of viruses as seen from the increase in CT values. Based on research by Scola BL et al (Viral RNA load as determined by cell culture as a management tool for discharge of SARS-CoV-2 patients from infectious disease wards) which states that CT values equal or above 34 do not excrete infectious viral particles. Our research data showed an increase in mean CT value equal or above 34 for the five groups after mouth rinsing and gargling for 3 days. Therefore we recommend mouth rinsing and gargling at least 3 days.

 Surprisingly we found that mouth rinsing and gargling with mineral water could increase the CT values as well. This is due to whirling water can mechanically wash out the virus and virus-infected cells from the oral cavity and pharynx. Mouth rinsing and gargling with mouthwash can disrupt the balance of flora in the oral cavity, therefore mouth rinsing and gargling with mineral water can be an alternative gargling procedure to prevent the attachment of the SARS-CoV-2 virus at an early stage that is safe for long-term use.

# Response

**Published:** 2024-05-27

**Authors:** Rosalina Tjandrawinata

**Affiliations:** Dental Materials, Universitas Trisakti, West Jakarta, Jakarta, Indonesia

Dear doctors,

 Thank you for the explanation. Now it is clear that whirling water can mechanically wash out the virus and virus-infected cells from the oral cavity and pharynx, and it is clearly shown in Table 2 that the content of the liquid does not make any significant difference to the result.

# Reviewer report: Reviewer response for version 1

**DOI:** 10.5256/f1000research.122496.r154813

**Published:** 2022-12-06

**Authors:** Muhammad Ruslin

**Affiliations:** 1 Department of Oral and Maxillofacial Surgery, Faculty of Dentistry, Hasanuddin University, Makassar, Indonesia

I appreciate the authors for presenting "The effects of mouth rinsing and gargling with mouthwash containing povidone-iodine and hydrogen peroxide on the cycle threshold value of Severe Acute Respiratory Syndrome Coronavirus 2: A randomized controlled trial of asymptomatic and mildly symptomatic patients". This will add more information to the existing literature on COVID-19-related cases.

 However, there are some mismatches between the conclusions and the data presented. In the whole manuscript, the authors never mentioned cases specific to oral and maxillofacial surgery; how come they finally recommend this rinsing solution/method for oral and maxillofacial surgery? From my point of view, it would be better to draw a specific conclusion related to the Covid-19 patients, since this is your main issue in the article. Based on the results of your study, what can you recommend for these patients?

 (please see the conclusion part, the second sentence, the sentence does not sound appropriate, please modify)

Is the work clearly and accurately presented and does it cite the current literature?

Yes

If applicable, is the statistical analysis and its interpretation appropriate?

I cannot comment. A qualified statistician is required.

Are all the source data underlying the results available to ensure full reproducibility?

Yes

Is the study design appropriate and is the work technically sound?

Yes

Are the conclusions drawn adequately supported by the results?

Partly

Are sufficient details of methods and analysis provided to allow replication by others?

Yes

Reviewer Expertise:

dentistry, oral and maxillofacial surgery

I confirm that I have read this submission and believe that I have an appropriate level of expertise to confirm that it is of an acceptable scientific standard, however I have significant reservations, as outlined above.

# Response

**Published:** 2023-01-30

**Authors:** Lilies Dwi Sulistyani

**Affiliations:** University of Indonesia, Indonesia

Thank you Prof Muhammad Ruslin. We appreciate your review and suggestions.

 In the introduction, we mentioned that this study departed from a large number of asymptomatic COVID-19 patients and mild symptoms which made a significant contribution to the spread of SARS-CoV-2 both to medical personnel and the people around the patient because there were no visible signs of active infection. These patients are often difficult to distinguish from healthy people, so there is a lack of awareness from health workers, especially those who perform procedures in the oral cavity, to apply transmission prevention protocols to these patients. We did not use samples for specific cases in the field of oral and maxillofacial surgery but instead used asymptomatic and mildly symptomatic COVID-19 patients. Reducing the amount of virus in the oral cavity and back of the throat before the medical procedure with mouth rinsing and gargling protocol is easy to perform. It can be applied in the field of oral and maxillofacial surgery. Not only oral and maxillofacial surgeons, but colleagues such as anesthesiologists, ENT, and others who perform any medical procedures in the oral cavity can also apply this mouth rinsing and gargling protocol as we stated in the second sentence of the conclusion.

# Response

**Published:** 2024-04-22

**Authors:** Lilies Dwi Sulistyani

**Affiliations:** University of Indonesia, Indonesia

Hi Prof Ruslin. Thank you for reviewing the article.

 I have modified the second sentence in the conclusion as per your suggestion.
